# The Biology and Pathobiology of Glutamatergic, Cholinergic, and Dopaminergic Signaling in the Aging Brain

**DOI:** 10.3389/fnagi.2021.654931

**Published:** 2021-07-13

**Authors:** Anna Gasiorowska, Malgorzata Wydrych, Patrycja Drapich, Maciej Zadrozny, Marta Steczkowska, Wiktor Niewiadomski, Grazyna Niewiadomska

**Affiliations:** ^1^Mossakowski Medical Research Institute, Polish Academy of Sciences, Warsaw, Poland; ^2^Nencki Institute of Experimental Biology, Polish Academy of Sciences, Warsaw, Poland

**Keywords:** glutamatergic, cholinergic, dopaminergic system, physiological aging, neurodegenerative diseases, therapeutic targets

## Abstract

The elderly population is growing worldwide, with important health and socioeconomic implications. Clinical and experimental studies on aging have uncovered numerous changes in the brain, such as decreased neurogenesis, increased synaptic defects, greater metabolic stress, and enhanced inflammation. These changes are associated with cognitive decline and neurobehavioral deficits. Although aging is not a disease, it is a significant risk factor for functional worsening, affective impairment, disease exaggeration, dementia, and general disease susceptibility. Conversely, life events related to mental stress and trauma can also lead to accelerated age-associated disorders and dementia. Here, we review human studies and studies on mice and rats, such as those modeling human neurodegenerative diseases, that have helped elucidate (1) the dynamics and mechanisms underlying the biological and pathological aging of the main projecting systems in the brain (glutamatergic, cholinergic, and dopaminergic) and (2) the effect of defective glutamatergic, cholinergic, and dopaminergic projection on disabilities associated with aging and neurodegenerative disorders, such as Alzheimer’s and Parkinson’s diseases. Detailed knowledge of the mechanisms of age-related diseases can be an important element in the development of effective ways of treatment. In this context, we briefly analyze which adverse changes associated with neurodegenerative diseases in the cholinergic, glutaminergic and dopaminergic systems could be targeted by therapeutic strategies developed as a result of our better understanding of these damaging mechanisms.

## Introduction

Aging is the process of progressive and usually slow degradation of a living organism. Understanding the physiological aging process in both animal models and humans is difficult because of the lack of well-defined aging indicators/factors and the lack of biomarkers indicating the onset of this process. In the central nervous system (CNS), aging involves the cognitive impairment, motor disorders, or emotional disturbances (Johnson et al., 2008), alterations that are the consequence of changes in the brain. Several studies in which rigorous quantitative methods have been used show that the number of neurons in the hippocampus, putamen, medial mammillary nucleus, hypothalamus, and the nucleus basalis of Meynert (NBM) seems to be stable through physiological aging (Freeman et al., 2008). Changes in aged brains include shrinkage of neurons in several areas, such as the neocortex, hippocampus, basal forebrain, and substantia nigra (SN) (Coleman and Flood, 1987; Fjell et al., 2014), and decreased dendritic length or complexity in certain brain areas (Jacobs et al., 1997; de Brabander et al., 1998) and loss of synapses (Presumey et al., 2017; Cobley, 2018; Wilton et al., 2019).

**FIGURE 1:**
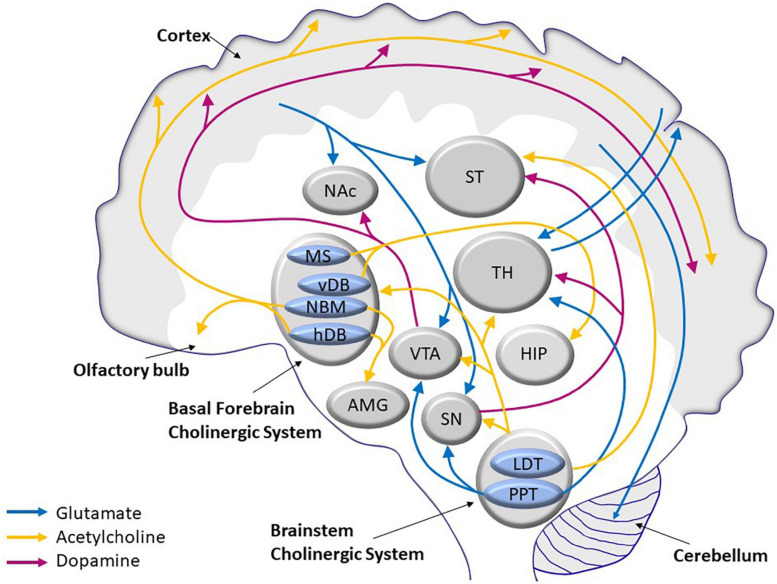
Scheme of the main glutamatergic, cholinergic, and dopaminergic neurocircuits and their convergence in the human brain. AMG, amygdala; HDB, horizontal limb of the diagonal band of Broca; HIP, hippocampus; LDT, laterodorsal tegmental nucleus; MS, medial septum; NAc, nucleus acccumbens; NBM, nucleus basalis of Maynert; PPT, pedunculopontine tegmental nucleus; SN, substantia nigra; ST, striatum; TH, thalamus; VDB, vertical limb of the diagonal band of Broca; VTA, ventral tegmental area.

The age-related morphological changes are accompanied by a decline in several neurotransmission pathways, namely, glutamatergic (Kumar and Foster, 2019), cholinergic (Henke and Lang, 1983; Shen and Barnes, 1996), and dopaminergic (Reeves et al., 2002; Luo et al., 2019) pathways. These three systems interact by stimulating or inhibiting activities of the others. The cholinergic system, which modulates pyramidal neurons in the cerebral cortex, and the glutaminergic system, which influences dopaminergic projection in the striatum (Figure 1), control important vital functions, such as cognitive and motor skills.

The cholinergic system is involved in memory and learning, and the impairment of its function due to loss of neurons or loss of their cholinergic phenotype compromises cognition and is regarded as the earliest event in Alzheimer’s disease (AD) etiology. In contrast, glutamatergic neurons are the most abundant in the CNS; and, consequently, their loss constitutes the greatest share in brain atrophy. Dopaminergic neurons, relatively scarce in number compared with other neuronal populations, are particularly prone to neurodegeneration, and their loss in the substantia nigra pars compacta (SNpc) causes motor impairment to the extent of immobilization in Parkinson’s disease (PD) in the absence of medication.

During physiological aging, all these systems undergo age-related changes resulting in continued although the modest loss of neurons, consistent with functional decline. However, thanks to neural plasticity, compensatory strategies are spontaneously developed and help to maintain sufficient cognitive capacity. The loss of functional independence stems rather from physical incapacitation caused by loss of mass and strength of skeletal muscles, driven probably by the age-related and unstoppable death of motoneurons, which even occurs in otherwise healthy elderly persons.

Pathological aging manifests itself in an increased rate of neuronal loss resulting in massive brain atrophy, accelerated decline in mental capacity, neurodegenerative disorders, and, finally, in complete dependence on round-the-clock care. The most common age-related neurodegenerative diseases are AD and PD. The clinical symptoms of AD are progressive loss of memory and cognitive impairment. In patients with AD, abnormal accumulation of amyloid β (Aβ) and tau proteins in senile plaques and neurofibrillary tangles may lead to astrocytosis and microgliosis. Furthermore, in cognitively normal aged people, senile plaques, granulovacuolar degeneration, and Hirano bodies are present but to a substantially lesser extent (Serrano-Pozo et al., 2011). PD is a progressive neurodegenerative disease associated with slow and continuous degeneration of dopaminergic neurons in SNpc and in the ventral tegmental area (VTA). The neurodegeneration within the nigrostriatal system is the dominant but not the only pathological process characterizing PD. The pathology of PD also includes changes in dopaminergic neurons in the mesocorticolimbic system and in the hypothalamic dopaminergic system. In the later stages of the disease, cholinergic (the basal nucleus of Meynert), serotonergic (raphe nuclei), and noradrenergic (locus coeruleus) systems are also affected (Peterson and Li, 2018; Liu, 2020). PD symptoms include both motor and non-motor symptoms (DeMaagd and Philip, 2015).

**FIGURE 2:**
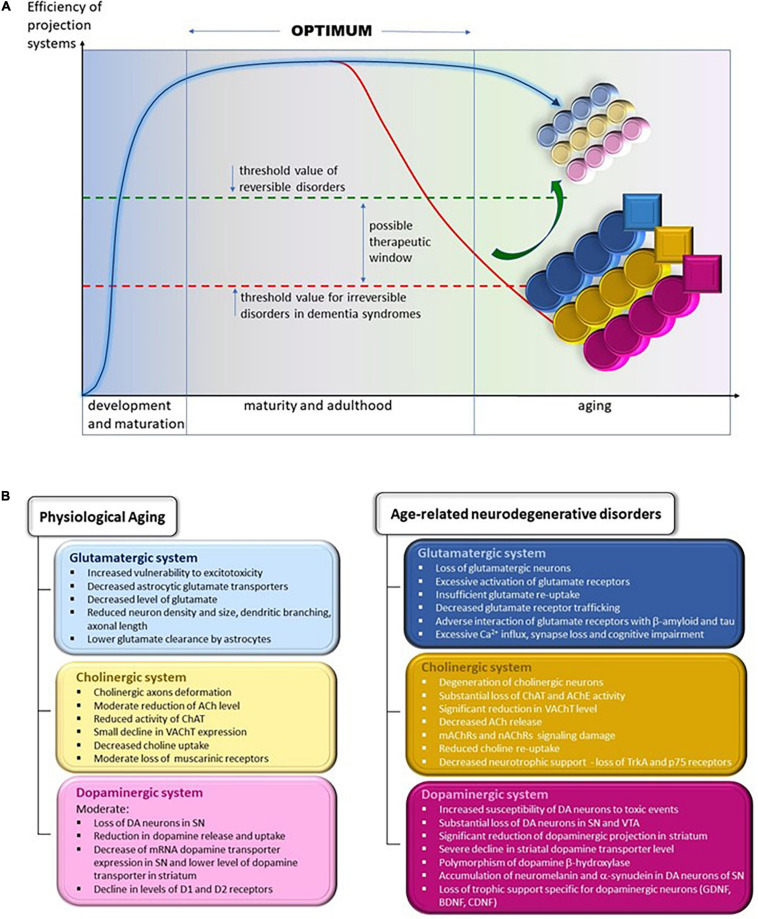
**(A)** Changes in the efficiency of projection systems during physiological aging and in age-related neurodegenerative disorders. Efficiency of projecting systems declines slowly during physiological aging because of the slow but progressive deterioration in various mechanisms affecting cholinergic, glutamatergic, and dopaminergic systems, symbolized by small, lightly colored circles; yellow, cholinergic; blue, glutamatergic; and pink, dopaminergic. The overall efficiency of these systems allows the elderly to remain fully independent in daily activities and to continue professional activities (threshold value of reversible disorders). We may assume that the accelerated worsening of the various process leads to their severe and extensive impairment, symbolized by larger and heavy colored circles. In addition, pathological processes appear that are not observed during physiological aging, symbolized by squares. The overall efficiency of projecting systems declines gradually, diminishing the functional independence and hampering professional activities. The overt decline calls for medical intervention, which helps to alleviate symptoms and probably may slow the progression of deterioration. At present, there are no therapies that reverse (symbolized by the green arrow) or even stop the progression of neurodegeneration. Thus, inevitably, functional regression continues up to complete dependence, necessitating round-the clock care until death. This situation is symbolized by being below the threshold value for irreversible disorders in the line for dementia syndromes line. **(B)** Specific changes in the structure and function of glutamatergic, cholinergic, and dopaminergic projection systems during physiological aging (left panel) and in age-related neurodegenerative diseases (right panel). ACh, acetylcholine; AChE, acetylcholine esterase; BDNF, brain-derived neurotrophic factor; CDNF, cerebral dopamine neurotrophic factor; ChAT, choline acetyltransferase; D1 and D2, dopaminergic receptors 1 and 2; DA, dopamine/dopaminergic; GDNF, glial cell line-derived neurotrophic factor; mAChRs, muscarinic acetylcholine receptors; nAChRs, nicotinic acetylcholine receptors; p75, low-affinity nerve growth factor receptor; SN, substantia nigra; TrkA, tropomyosin receptor kinase A/high-affinity nerve growth factor receptor; VAChT, vesicular acetylcholine transporter; VTA, ventral tegmental area.

In this review, we have compared the changes in the glutamatergic, cholinergic, and dopaminergic systems during physiological and pathological aging in humans and in mouse and rat models of AD and PD. We aim to show that many changes in these three systems, although qualitatively similar in physiological and pathological aging, differ in intensity and advancement. We will identify the changes that are not present in physiological aging and are most probably irreversible, such as substantial death of selected neuron populations and loss of motor and cognitive functions in neurodegenerative diseases (Figures 2A,B). To develop future treatment strategies, we suggest that it is important to determine whether and how the gradual age-related changes in normal aging are accelerated in disease conditions.

## Glutamatergic System

### Glutamate Signaling Pathways in Multiple Brain Functions and Synaptic Plasticity

Glutamate is the most common neurotransmitter in the CNS. Almost 40% of all neurons are classified as glutamatergic, and more than 90% of all neurons have glutamate receptors. Most glutamatergic neurons are located in the frontal cortex. There are five major cortical glutamate pathways (Figure 1): (1) the cortico-cerebellar pathway that controls glutamate release; (2) the cortico-striatal and cortico-accumbens pathways that participate in the cortico-striatal-thalamic loops; the (3) thalamo-cortical and (4) cortico-thalamic pathways; and finally, the (5) cortico-cortical pathway (not shown in Figure 1), where glutamatergic neurons communicate with each other. The cortico-cerebellar, cortico-striatal, and cortico-thalamic pathways send projections to the subcortical structures and regulate glutamate neurotransmission (Stuber et al., 2010; Reiner and Levitz, 2018). Because of its extensive projection circuits, glutamate is involved in learning, memory formation and storage, and synaptic plasticity (Mayer and Westbrook, 1987). The ability to induce long-term potentiation (LTP) or long-term depression (LTD) in the hippocampus by glutamatergic receptors, both ionotropic and metabotropic, determines synaptic plasticity (Collingridge et al., 2010). Synaptic plasticity is often calcium-dependent and requires alteration of the actin cytoskeleton in dendrites and local mRNA translation of synaptic proteins (Sapoznik et al., 2006; Murakoshi and Yasuda, 2012; Bosch et al., 2014).

#### Receptors

There are two types of glutamatergic receptors: ionotropic (iGluR) and metabotropic (mGluR). Ionotropic receptors are ligand-gated ion channels activated by glutamate. Binding of glutamate to such a receptor causes excitation of the neuron by depolarization. The vast majority of glutamatergic transmission is mediated by the ionotropic α-amino-3-hydroxy-5-methyl-4-isoxazolpropionic acid and *N*-methyl-D-aspartate receptors (AMPARs and NMDARs, respectively). The best-known glutamate receptor is an NMDAR. This ionotropic receptor may be synaptic or extra-synaptic. In addition to glutamate, it can also bind glycine. Upon deactivation, because of low voltage and weak stimulation, Mg^2++^ blocks the ion channel. Activation of synaptic NMDARs is involved in LTP signaling, and activation of extra-synaptic NMDARs is associated with LTD and excitotoxicity (Hardingham and Bading, 2010). Activation of NMDARs requires the binding of two molecules of glutamate or aspartate and two glycines (Laube et al., 1997). Opening the channel causes the influx of calcium ions into the cell. The synaptic signal is then amplified by the Ca^2+^/calmodulin-dependent cascade activation of protein kinase II (CaMKII). Physiological stimulation of synaptic NMDARs is essential for the survival of the neuron. This is related to NMDAR neuroprotective function – suppression of apoptosis and activation of transcription factors promoting survival, such as cyclic AMP response element (CRE)-binding protein (CREB) (Monti and Contestabile, 2000; Kolarova et al., 2003; Hardingham and Bading, 2010). However, an NMDAR is also responsible for glutamate toxicity. Overstimulation of NMDARs can cause an excessive influx of Ca^2+^ into the neuron and, thus, cell death. Long-term imbalance in NMDAR signaling leads to neurodegenerative diseases and acute cell damage (Hardingham and Bading, 2010). In turn, weak stimulation of extra-synaptic NMDARs causes a slight increase in Ca^2+^ levels in the postsynaptic compartment and the release of phosphatases that mediate LTD induction (Luscher and Malenka, 2012).

The AMPAR is another receptor that binds glutamate to transmembrane ion channels. It often coexists in the same synapse as NMDAR and can enhance its activity. Glutamate binding to AMPARs (Twomey et al., 2017) opens ion channels and causes an influx of Na^+^ that initiates depolarization. AMPARs respond to stimulation even weaker than that triggering evoked potential, resulting in weak depolarization of the postsynaptic membrane. Another group of ionotropic receptors are kainate receptors, which are functionally very similar to AMPARs (Pinheiro and Mulle, 2006).

Metabotropic glutamate receptors belong to the family of G proteins; and mGluRs are widespread in the CNS, implying their involvement in many functions of the brain. They form three groups of receptors. Group 1 receptors (mGluR1 and mGluR5) are postsynaptic receptors that, like NMDARs, mediate direct excitatory synaptic transmission, promote Ca^2+^ inflow, and lead to the activation of protein kinase C (PKC), which increases the likelihood of NMDAR channel opening (Riedel et al., 2003; Niswender and Conn, 2010). Group 2 (mGluR2 and mGluR3) and Group 3 receptors (mGluR4, mGluR6, mGluR7, and mGluR8) are found mainly presynapticaly in glutamatergic and GABAergic neurons. They down-regulate adenylate cyclase (cAMP), inhibit Ca^2+^ channels, and activate K^+^ channels (Niswender and Conn, 2010; Ribeiro et al., 2017). In addition, they act as autoreceptors that inhibit the release of glutamate or gamma-aminobutyric acid (GABA). All groups of metabotropic receptors may have neuroprotective properties and promote cell proliferation or activate a survival-promoting signaling cascade (Kingston et al., 1999a, b; Ribeiro et al., 2017). However, the mGluR5 present in astrocytes may be involved in astrocyte dysfunction or degeneration (Paquet et al., 2013). This may cause impairment in glutamate turnover during aging and age-related neurodegenerative diseases (Palmer and Ousman, 2018).

#### Glutamate Turnover – Close Cooperation of Neurons and Astrocytes

Central nervous system glutamate is located almost exclusively inside cells. Free extracellular glutamate level is tightly regulated by astrocytes to ensure proper transmission. Astrocytes are essential in the synthesis and reuptake of glutamate. Released glutamate molecules, which do not bind to receptors, are removed from the extracellular space by transporters present in the astrocyte membrane, preventing overstimulation of neurons by glutamate (Paulsen et al., 1987). The astrocytic glutamate transporters are excitatory amino acid transporter (EAAT) isoforms 1 and 2. The mouse homologues are glutamate aspartate transporter 1 (GLAST1) and glutamate transporter 1 (GLT-1). Almost 80% of the glutamate taken up from the extra-synaptic space is converted into glutamine by glutamine synthetase (Farinelli and Nicklas, 1992). Glutamine can be taken up again by the presynaptic neuron. Inside the neuron, glutamine is converted to glutamate by phosphate-activated glutaminase (PAG), which completes the glutamate turnover cycle.

Most of the glutamate comes from the glutamine–glutamate cycle (Shank et al., 1985). In addition, glutamate can also be produced from α-ketoglutarate (αKG). αKG is an intermediate in the tricarboxylic acid (TCA) cycle, which means that glutamate can be synthesized from glucose (McKenna, 2007). Synthesis in this way requires pyruvate carboxylase, an enzyme that is not present in neurons but is present in astrocytes. However, this synthetic pathway is not very efficient. Some glutamate is lost because of conversion into lactate, glutathione, or purines (Shank et al., 1985; Sonnewald et al., 1993).

### Physiological Aging of Glutamatergic System

Neurons in the aging brain are particularly susceptible to excitotoxicity. For this reason, malfunction of the glutamatergic system, which may readily lead to excitotoxicity, can be particularly dangerous for the nervous system.

#### Number of Glutamatergic Neurons

In animal studies, a decreased number of glutamatergic neurons were observed in aged rats, mostly in the frontal and cerebral cortices (Heumann and Leuba, 1983), and subtle changes in synaptic composition and function in the hippocampus were reported (Shi et al., 2007). The loss of these neurons was not very severe and may reflect the normal physiological extinction of nerve cells with age.

#### Glutamate Level and Metabolism

Studies on rats indicate that there is no change in both glutamate and glutamine syntheses, and that glutamate turnover is unchanged with age. This is supported by the level and activity of astrocytic glutamine synthetase, which similarly does not change with age (Segovia et al., 2001a). Saransaari and Oja (1995) found that glutamate level generally increased during adolescence and decreased with age in mice. Nevertheless, glutamatergic signaling is not disrupted despite decreased glutamate level, probably because glia and unaffected neurons compensate for this deficiency (Porras and Mora, 1995). Neuronal plasticity is retained with age by being able to increase the intracellular release of Ca^2+^ from cytoplasmic stores (Sapoznik et al., 2006). Results obtained from studies on mice and rats showed a decrease in the level of glutamate in the cortex during aging (Strolin-Benedetti et al., 1991; Saransaari and Oja, 1995; Jing et al., 2016). In contrast, Stephens et al. (2011) showed heterogeneity of hippocampal glutamatergic neurotransmission in aging F344 rats. The resting level of glutamate in the extracellular space was unchanged with aging; however, stimulus-evoked glutamate release and clearance capacity was enhanced in the dentate gyrus of aged rats. On the other hand, most *in vitro* and *in vivo* studies have not shown changes in glutamate release during aging. This stable level of glutamate release in aging organisms has been observed in most of the brain areas of NMRI mice and Fisher and Wistar rats (reviewed in Segovia et al., 2001b). The lack of change in glutamate release may be due to compensatory changes in glutamate uptake. Reduced glutamate uptake and a loss in the number of high-affinity glutamate transporters in glutamatergic terminals of 24-month-old aged rats have been described (Vatassery et al., 1998).

Glutamate levels decline in the hippocampus, anterior cingulate gyrus, and other cortical areas during aging (Schubert et al., 2004; Kaiser et al., 2005; Chang et al., 2009; Hädel et al., 2013; Huang et al., 2017). A magnetic resonance spectroscopy (MRS) study on healthy young and old patients revealed that the glutamate-glutamine-glutamate turnover cycle is also affected in the anterior cingulate cortex and hippocampus. In addition, it has been shown that the density of glutamatergic neurons and their metabolic activity are reduced in cerebral lobes and in the cerebellum of healthy elderly people (Ding et al., 2016; Huang et al., 2017). A meta-analysis of glutamate neurometabolites also confirms a decline in the glutamatergic system status in the elderly compared with younger people. Glutamate concentrations were significantly lowered in older compared with those in younger adults, whereas the concentration of glutamine was significantly higher in older individuals (Roalf et al., 2020). The higher level of glutamine in the elderly group is probably due to an imbalance of the neural-astroglial mechanism regulating glutamate metabolism; and glutamate neurometabolites are found mainly in neurons, whereas glutamine is found in glial cells. Age-related impairment of the glutamatergic system also includes the decreased size of glutamatergic neurons, fewer dendritic branches, and shortened axon length, and decreased glutamate neurotransmission, which may contribute to cognitive impairment (Drachman, 2006).

#### Expression of Glutamatergic Receptors

The most significant and consistent finding is the age-related decrease in the density of glutamatergic NMDA receptors in the cortical areas, striatum, and hippocampus of Long–Evans and Fischer 344 rats (Nicolle et al., 1996; Mitchell and Anderson, 1998). It seems that aging animals are more sensitive to a higher concentration of glutamate in the synaptic cleft, as shown in senescence-accelerated mice (SAM-P/8) (Kitamura et al., 1992) and in different strains of mice and rats (Segovia et al., 2001b). Data on age-related changes in the AMPAR expression are more ambiguous. In C57Bl and BALB/c mice, a decrease in glutamatergic AMPAR densities with increasing age (3–30 months) in the frontal and parietal cortices and hippocampal CA1 region has been reported (Magnusson and Cotman, 1993), whereas in rats, the AMPAR density in the cortical areas did not change with age (Cimino et al., 1993). No age-related changes in metabotropic receptor density were observed in rats, as well as no alteration in the binding of glutamate and in responses mediated by these receptors were reported (Jouvenceau et al., 1997; Magnusson, 1997). The EAAT level decreases with aging in Sprague–Dawley rats, which may facilitate glutamate toxicity (Potier et al., 2010; Pereira et al., 2017). Decreased glutamate transporter activity associated with increased excitotoxicity and neurodegeneration was observed in the brains of patients with AD, supporting the possibility that abnormal functioning of this system might be induced by increased stimulation of glutamatergic receptors (Masliah et al., 1996).

In summary, the glutamatergic system function does not appear to be significantly reduced in aging rodents and humans. Likewise, there is a slight downward trend in glutamate levels. The stability of the glutamatergic system may be the result of a balance between glutamate release and reuptake provided by a compensatory activity from astroglia. The age-related decline in glutamate appears to affect neurons rather than astrocytes. An increase in the number and/or activity of astrocytes during aging (Clarke et al., 2018) may compensate for the insufficient neuronal reuptake of glutamate. On the other hand, there are studies that have shown that aging astrocytes are less able to remove glutamate and convert it to glutamine and, thus, may be involved in excitotoxicity (Palmer and Ousman, 2018).

Understanding the physiological aging process in both animal models and humans is difficult because of the lack of well-defined aging indicators/factors and the lack of biomarkers for the onset of this process. These limitations need to be taken into account when interpreting the results.

### Age-Related Disturbances of Glutamatergic Signaling in the Pathogenesis of AD and PD

Glutamate seems to play a pivotal role in the etiology of AD and PD, because of its abundance in brain tissue and, in part, because it is at the crossroads of multiple metabolic pathways. It has been shown that if the balance of glutamate turnover is disrupted, the perturbation of glutamate neurotransmission has severe consequences, leading to the onset of neurodegenerative diseases (reviewed by Bukke et al., 2020; Iovino et al., 2020; Wang J. et al., 2020). Understanding the role of the glutamatergic system in the pathophysiology of AD and PD may allow the development of improved therapeutics for these neurodegenerative disorders.

#### Loss of Glutamatergic Neurons in AD

Glutamatergic pyramidal neurons are very sensitive to oxidative stress and to the excitotoxic effects of overstimulation. Hypersensitive neurons have a higher energy requirement; however, disrupted glutamatergic transmission causing excessive Ca^2+^ influx affects the two most important energy-producing organelles in the cell-mitochondria and the endoplasmic reticulum (Lan et al., 2014; Muddapu et al., 2020). Long-lasting oxidative stress and ionic imbalance may contribute to the loss of glutamatergic neurons in neurodegenerative diseases, such as AD or PD (Chen et al., 2013).

In patients with AD, there is evidence of a loss of glutamatergic neurons, especially in the neocortex (layers III and IV) and the CA1 region of the hippocampus (West et al., 1994; Gómez-Isla et al., 1996; Simić et al., 1997; de Toledo-Morrell et al., 2000; Price et al., 2001; von Gunten et al., 2006).

#### Impaired Glutamatergic Recycling and Signaling in AD

Glutamatergic neurons terminals are affected at an early stage in a mouse model of AD, produced by the intracerebral administration of soluble Aβ1–42 (Canas et al., 2014). Moreover, the glutamate recycling system appears to be severely impaired (Wang and Reddy, 2017). There is evidence that the toxic Aβ has a strong influence on the glutamatergic system. *In vitro* studies on cultured hippocampal astrocytes of Sprague–Dawley rats indicate that Aβ can decrease the uptake of glutamate from the synaptic cleft, and, thus, enables greater glutamate availability and activates the signaling cascades responsible for neuronal edema (Parpura-Gill et al., 1997; Fernández-Tomé et al., 2004). Aβ may also contribute to glutamate release by interacting with presynaptic proteins, such as synaptophysin or synaptotagmin, as shown in rat cortical neuronal cultures (Jang et al., 2014). Moreover, Aβ appears to directly modulate NMDAR function. It is also responsible for blocking the binding of glutamate receptor co-agonists or antagonists and loss of synapses in the SN/neuroblastoma hybrid cell line (MES 23.5) (Le et al., 1995), in pyramidal neurons of the organotypic hippocampal slices (Shankar et al., 2007), and in cultured cerebellar cells (Kawamoto et al., 2008). Aβ can regulate the functional state of NMDARs through synaptic proteins, such as PSD-95, in hippocampal neuronal cultures (De Felice et al., 2007). In addition, Fuchsberger et al. (2019) showed that oral administration of monosodium glutamate impairs memory through an increased accumulation of Aβ and phospho-tau and decreased AMPAR signaling and LTP blocking in APP/PS1 mice with amyloidopathy.

Changes in glutamatergic synapses are accompanied by altered calcium dynamics in dendrites and dendritic spines, leading to activation of calcineurin (Shankar et al., 2007; Cavallucci et al., 2013). Elevated Ca^2+^ levels may also contribute to the phosphorylation of tau and ubiquitin, proteins whose abnormal forms are present in AD (Mattson, 1992). Another consequence of Ca^2+‘^ influx is slow swelling and degeneration of neurons in murine cortical cell cultures (Koh and Choi, 1991). Abnormal calcium signaling activates catabolic enzymes that induce the production of reactive oxygen species (ROS) and reactive nitrogen species (RNS), which leads to damage of the cytoskeleton and cell membrane in cultured hippocampal neurons (Mattson, 1992).

Glutamatergic transmission and neuronal excitability are both modulated by mGluR. In AD, mGluR and Aβ interact with each other and can influence the severity of the disease. Group 1 mGluRs appear to influence Aβ toxicity (Chen et al., 2013). The role of group 2 mGluRs is rather ambiguous. On one hand, they may contribute to the production and release of Aβ (Kim et al., 2010); and on the other, they appear to exert a neuroprotective effect (Bruno et al., 1997). Group 3 mGluRs show mainly neuroprotective effects, as their main function is to reduce glutamate release (Bruno et al., 2000; Ribeiro et al., 2017). The metabotropic mGluR5 receptor appears to be a promising therapeutic target. Studies have shown that modulation of this receptor can reduce behavioral deficits, glutamate signaling disorders, and tau pathology in APPswe/PS1dE9 (APP/PS1) mice (Haas and Strittmatter, 2016).

There is also an association between defective tau phosphorylation and glutamate receptors, mediated by PSD-95 synaptic proteins and Fyn kinase. They mediate a mutual toxic interaction of Aβ and tau at synapses and activate the signaling cascade, leading to excitotoxicity and degeneration of neurons (Ittner et al., 2010; Revett et al., 2013; Hu et al., 2014).

It is postulated that in the late stage of the disease, tau pathology may cause irreversible changes in synaptic function (reviewed by Benarroch, 2018). Disruption of dendritic transport results in a reduction in the transport of glutamate receptors and proteins anchoring it in synapses and a reduction in the number of mitochondria in synapses of rTg4510 mice (Hoover et al., 2010) and in mice expressing mutant human presenilin 1, PS1 (M146L) as well as mice carrying the double mutation of human amyloid precursor protein APP (Tg2576) and PS1 (M146L) (Trushina et al., 2012). Phosphorylation of tau-specific domains may also induce excessive microtubule stability (impairment of dynamic instability, which is a physiological feature of microtubules) in dendrites, which may impair synaptic plasticity (reviewed by Brandt and Bakota, 2017). Interestingly, recent studies have shown that increased glutamate reuptake decreased tau pathology in a mouse model of AD [rTg(TauP301L)4510] (Hunsberger et al., 2015a, b) and in a transgenic *Drosophila* model of AD (Kilian et al., 2017). The data also suggest that as the disease progresses, tau accumulation in astrocytes may contribute to increased conversion of glutamate to GABA (Quevenco et al., 2019) and, thus, may prevent glutamate toxicity in mouse models of tauopathy (Crescenzi et al., 2017; Hebron et al., 2018).

In humans, a lower level of the vesicular glutamate transporter (VGluT) (Kirvell et al., 2006) and astrocytic EAAT dysfunction (Scott et al., 2011) has been reported. Glutamatergic neurotransmission decreases with physiological aging, but in patients with mild cognitive impairment (MCI) or AD, these disorders are more acute and correlate with dysfunctions in the glutamatergic system (Kantarci, 2007; Riese et al., 2015). Some researchers have postulated that reduced glutamatergic markers may be a good diagnostic tool for the early diagnosis of AD (Huang et al., 2017).

Changes in synaptic iGluRs and mGluRs are among the many pathological alterations observed in the development of AD. The reduction in the number of synapses containing glutamatergic receptors and dendritic branching dystrophy occurs in the early stage of AD and may affect the severity of the disease (Arendt, 2009; Pozueta et al., 2013; Benarroch, 2018). Excessive Ca^2+^ influx, which causes pathological signaling and gradual reduction of synaptic function and ultimately cell degeneration, was observed in patients with AD. Changes in Ca^2+^ influx correlated with memory impairment in patients with AD (Jansone et al., 2016). Memantine, which is a NMDAR antagonist and drug used in the treatment of AD, reduces the toxicity caused by excessive Ca^2+^ in humans (Lipton, 2005; Matsunaga et al., 2015; Kishi et al., 2017; Kabir et al., 2019).

#### Hyperactivity in Glutamatergic Signaling in PD

In patients with Parkinson’s disease (PD), the loss of dopaminergic neurons in the substantia nigra (SN) and the subsequent deficiency of dopamine (DA) in the striatum lead to the excessive activation of glutamatergic projection to the globus pallidus and to the pars reticularis of SN. Glutamate antagonists appear to have anti-parkinsonian therapeutic activity (Greenamyre and O’Brien, 1991; Schmidt et al., 1992; Blandini et al., 1996; Blandini, 2010). In PD, disturbance of glutamate homeostasis and excitotoxicity are associated with excessive NMDAR activation (Wang J. et al., 2020; Trudler et al., 2021) and insufficient glutamate reuptake in the striatum (Calon et al., 2003; Iovino et al., 2020). In early PD, this glutamatergic hyperactivity may compensate for the loss of neurons in SN (Ampe et al., 2007; Shimo and Wichmann, 2009), but as the disease progresses, it causes impairment of the striatal signaling loop. In addition, VGluT1 and VGluT2 levels are altered in specific regions of the Parkinson’s brain. VGluT1 and VGluT2 expression was increased in the putamen, whereas VGluT1 was dramatically decreased in the prefrontal and temporal cortices of patients with PD (Kashani et al., 2007). Interestingly, a growing body of research indicates an important role for mGluR in motor control, as it produces a direct excitation and disinhibition of GABAergic projection neurons in the substantia nigra pars reticulata (Awad et al., 2000; Marino et al., 2001). Group 1 mGluRs respond to dopaminergic stimulation in experimental models of PD (Dekundy et al., 2006), Group 2 mGluRs reduce glutamate release (Picconi et al., 2002) and modulate synaptic inputs and calcium signals in striatal cholinergic interneurons (Pisani et al., 2002), and Group 3 mGluRs reduce both GABAergic and glutamatergic transmission in the rat substantia nigra pars reticulata and globus pallidus (Wittmann et al., 2001; Matsui and Kita, 2003). Persistently high glutamate levels can modulate/suppress DA evoked release by activating the Group 1 mGluRs in SNpc DA terminals.

In summary, chronically increased excitatory glutamatergic signaling, even if moderate in physiological aging, tends to induce excitotoxicity, leading to neuronal degeneration. In several neurodegenerative diseases, such as AD and PD, ample evidence suggests that glutamatergic dysregulation is an important contributor to disease pathology, although the molecular basis for this may be different for each disease and may reflect multiple pathways leading to disease. Contrary to physiological aging, disorders of the glutamatergic system in disease states are associated with severe deficiency of information transmission in the neural network and impairment of cognitive processes (Lewerenz and Maher, 2015; Figure 2B).

## Cholinergic System

### Role of Cholinergic Projection in Cognitive Function

The cholinergic basal forebrain is composed of a collection of magnocellular hyperchromic neurons located within the medial septum (MS), vertical and horizontal limbs of the diagonal band of Broca (vDB and hDB respectively), and basal magnocellular nucleus (NBM). These neurons in the septal/diagonal band complex and in NBM provide the major cholinergic innervation to the hippocampus and the entire neocortical mantle, respectively (Mesulam et al., 1983; Hedreen et al., 1984; Figure 1).

#### Receptors

The primary neurotransmitter of the cholinergic system, acetylcholine (ACh), acts as a neuromodulator and takes part in control of cortical activity. The impact of ACh on the cortical circuit depends on the expression of its specific receptors and the concentration of the neurotransmitter. There are five cholinergic muscarinic receptor subtypes (M1–M5), all of them being expressed in the CNS but at different levels and in different locations. For example, M1 and M3 appear to be the most abundant muscarinic receptors expressed in the hippocampus and entorhinal cortex in adult mouse, whereas M5 is poorly expressed. The M1–M5 receptors can be subdivided into two major functional classes according to their G-protein coupling preference. The M1, M3, and M5 receptors selectively couple to G-proteins of the Gq/G11 family, whereas the M2 and M4 receptors preferentially activate Gi/Go-type G-proteins. Coupling ACh through the first group (M1, M3, and M5) but not through the second group results in an increase in intracellular calcium (Lanzafame et al., 2003).

Nicotinic acetylcholine receptors (nAChRs) are a family of transmembrane neurotransmitter receptors that play critical functions in the central and peripheral nervous systems (Albuquerque et al., 2009). They are also found in neuromuscular junctions. Nicotinic receptors consist of five subunits and combine into heteromeric and homomeric pentamers (Dani, 2015). In mammals, 16 subunits have been identified, and marked with Greek letters and then Arabic numbers – α1–α7, α9–10, β1–β4, γ, δ, ε. Neurons and muscle cells contain nicotinic receptors composed of distinct subunits, which result in the classification of nAChR into neuronal and muscle types, e.g., α4β2- and α7-nAChR (neuronal type) and α1β1γδ-nAChR (muscle type). nAChRs mediate some actions of the neurotransmitter ACh in the neuromuscular junction of the autonomic ganglia and at selected synapses in the brain and spinal cord. Neuronal nAChRs influence the release of the neurotransmitter, interacting with the signaling pathways of secondary messengers and controlling the flow of Ca^2+^ (Zoli et al., 2015).

#### Modulation of Synaptic Plasticity and Cognitive Processes

ACh can alter the activity of pyramidal neurons through rapid inhibition followed by slow depolarization. The rapid inhibition is partly mediated by nAChRs and mAChRs, which stimulate cortical GABAergic interneurons. In contrast, slow depolarization is moderated by M1 mAChRs, activation of which leads to the closure of M-type potassium channels in pyramidal neurons. Moreover, ACh increases glutamate release by binding to α4β2 nAChRs at the endings of thalamo-cortical projections in the sensory and associative cortices. On the other hand, activation of mAChRs on parvalbumin-positive interneurons lowers GABA release and reduces the inhibition of pyramidal cells. Activation of inhibitory M2 mAChRs at the axonal ends of pyramidal cells inhibits cortico-cortical transmission (Picciotto et al., 2012; Obermayer et al., 2017). The activation or inhibition of nicotinic and muscarinic receptors in the cholinergic projection system is responsible for the control of autonomic processes, sleep, wakefulness, and cognitive processing.

Synaptic plasticity is a mechanism connected with learning and memory, which can be modulated by ACh in hippocampus. Activation of cholinergic signaling via α7 nAChRs induces LTP and suppresses LTD induction, influencing synaptic plasticity in mouse hippocampal slices (Nakauchi and Sumikawa, 2012, 2014). Administration of carbachol, a cholinergic agonist, enhanced LTP in the CA1 region of the rat hippocampus (Blitzer et al., 1990).

The LTP phenomenon may underlie spatial memory formation, which is mediated by cholinergic signaling from the MS and vDB nuclei to the hippocampus. In memory tests performed in rats, ACh levels were elevated in the hippocampus (Mitsushima et al., 2013) and in the fronto-parietal cortex during a continuous attention task (Arnold et al., 2002). Damage to or inhibition of the activity of MS and vDB neurons leads to impairment of learning and memory in rats (Mizumori et al., 1990; Markowska et al., 1995). In addition, injections of scopolamine, a muscarinic receptor antagonist, into the rat hippocampus showed lower scores on a spatial discrimination task. This suggests a role for mAChRs in spatial learning (Carli et al., 1999; de Bruin et al., 2011). Similarly, in healthy adults, scopolamine causes memory deficits (Thomas et al., 2008). Healthy aging humans and animals perform worse in hippocampus-related learning and memory tasks as compared with younger adults, and nicotine administration remedies partly or completely this deficit (Zeid et al., 2018). In aging rodents, hippocampal LTP facilitation and immediate improvement of spatial memory is caused by both acute and chronic types of nicotine administration (Levin and Torry, 1996; Srivareerat et al., 2011). The acute and chronic nicotine types of administration improve cognitive performance in patients suffering from neurodegenerative disorders. It was found that relatively short-term nicotine patch exposure improved learning and memory in patients with probable AD, and that this improvement persisted throughout the washout period (Wilson et al., 1995).

#### Dependence on the Availability of Neurotrophic Factors

The differentiation, survival, and function of basal forebrain cholinergic neurons (BFChN) are dependent upon the actions of nerve growth factor (NGF) and its high-affinity receptor tyrosine kinase (TrkA) and the low affinity receptor p75^NTR^ (Huang and Reichardt, 2001; Skaper, 2018). These receptors are produced in BFChN and transported to their projection sites (Sobreviela et al., 1994). The target areas of cholinergic neurons (cortex and hippocampus) contain the highest level of NGF protein and NGFmRNA in the brain. Furthermore, the vast majority of mRNA and protein for both TrkA and p75^NTR^ NGF receptors are expressed by BFChN in both rats and humans (Sofroniew et al., 2001; Niewiadomska et al., 2011). By combining *in situ* hybridization detection of trkAmRNA with immunocytochemical detection of protein, it was determined that the TrkA receptor and its mRNA was also detected in other regions of the brain, namely, paraventricular nucleus of the thalamus, interpeduncular nucleus, prepositus hypoglossal nucleus, vestibular nuclei, raphe obscuris, cochlear nucleus, sensory trigeminal nuclei, hippocampus, and gigantocellular as well as paragigantocellular neurons in the medullary reticular formation (Gibbs and Pfaff, 1994; Lee et al., 1998; Bhattacharyya and Mondal, 2013). In contrast to TrkA, p75^NTR^ was found only in a minority of NGF-responsive cell populations (Holtzman et al., 1995).

### Physiological Aging of Cholinergic System

The early accepted view that there is a significant loss of cholinergic cells during physiological aging is now questioned. It is assumed that cholinergic neurons of the basal forebrain undergo moderate degenerative changes during aging, resulting in cholinergic hypofunction, leading to memory deficits that progress with age. Significant neuronal cell loss has been found rather in pathological aging, such as AD, while normal aging is accompanied by a gradual loss of cholinergic function due to dendritic synaptic and axonal degeneration, as well as a reduction in trophic support, such as the one mediated by NGF. As a consequence of these changes, there is only a loss in the cholinergic phenotype of the basal neurons and not in their atrophy (Schliebs and Arendt, 2011).

#### Animal Studies

From studies with aged rats as a model of physiological aging (Niewiadomska et al., 2000, 2002) provide evidence for only moderate deterioration of the cholinergic system with age. Age-related impairment of the rat cholinergic system is related to the loss in the basal cholinergic phenotype of neurons, as measured by choline acetyl transferase (ChAT) and the TrkA expression, rather than to the acute degeneration of neuronal cell bodies. This assumption was confirmed by the restoration of the cholinergic phenotype of basal forebrain neurons with the NGF treatment of old rats, increasing their number and restoring normal morphology.

#### Human Studies

Studies on non-demented young and old people have shown that axons in the brain of youngest subjects were almost homogenous in diameter and thin with small, fine varicosities. In older people, axons present some deformations, but the number of such abnormalities was increased significantly in very old subjects. There were no Aβ deposits in the basal forebrain cholinergic neurons in young people, but they appeared in small numbers in the brains of older people. On the other hand, in basal forebrain areas, tau-containing filamentous tangles appeared in young people, and their number increased with age (Geula et al., 2008). In addition, a decrease in the expression of the calbindin-D28K gene in the striatum and the NBM was observed with aging, which likely disrupts the Ca^2+^-buffering capacity and leads to hypofunction or atrophy of some cholinergic neurons (Geula et al., 2003; Ahmadian et al., 2015).

There is also a decrease in ACh synthesis with age in the cortex (Schliebs and Arendt, 2011). One of the limiting steps in the synthesis of ACh is the concentration of choline, which is transported back into the cell by high affinity choline uptake. Brain choline uptake is reduced in the elderly compared with younger people (Decker, 1987). Choline and acetyl coenzyme A are substrates for ChAT, which carries out the synthesis of ACh. In mentally healthy people, ChAT activity is reduced with age, particularly in the hippocampus and in the cortex and caudate nucleus (Perry et al., 1977; Decker, 1987). However, another study found no statistical difference between older and younger patients in ChAT activity in the cortex and the caudato-putamen (Wilcock et al., 1982; Nagai et al., 1983; Gilmor et al., 1999; Contestabile et al., 2008). These data are in agreement with those from vesicular acetylcholine transporter (VAChT) studies, which showed only a slight age-dependent decrease in VAChT binding to the cell membrane of presynaptic cholinergic terminals in the cortex and hippocampus (Kuhl et al., 1996). It is possible that age-related cholinergic dysfunction is the result of decreased choline uptake and ACh turnover (Cohen et al., 1995; Katz-Brull et al., 2002), which may lead to axonal degeneration and synaptic loss but not neurodegeneration (Grothe et al., 2013).

An *in vivo* single photon emission computed tomography (SPECT) study demonstrated a decrease in nicotinic receptor availability dependent on age (Mitsis et al., 2009). Studies in human brains have shown that with age, the binding of nicotine to high-affinity receptors in the hippocampus, entorhinal cortex, and the frontal and temporal cortices is reduced with advancing age (Court et al., 1997; Marutle et al., 1998; Hellström-Lindahl and Court, 2000). Moreover, in the human frontal cortex, the mRNA expression of α4 and β2 nAChRs subunits also declines with age (Tohgi et al., 1998a), but in the hippocampus and putamen only the level of β2 nAChR mRNA is significantly reduced (Tohgi et al., 1998a, b). Muscarinic receptor binding of cholinergic ligands is also reduced with age in the subicular complex and entorhinal cortex (Court et al., 1997), caudate nucleus, putamen, frontal cortex and hippocampus (Rinne, 1987). The binding decline in the subicular complex and entorhinal cortex concerns M1 and M3+4 receptor subtypes. Detailed analysis revealed that the reduced ligand binding was not due to a decline in the number of receptors. It was generally shown that during healthy aging and the development of neurodegenerative diseases, the overall mAChR level appears to be preserved or moderately reduced in the neocortex and hippocampus (Palacios, 1982; Smith et al., 1988; Lebois et al., 2018).

### Age-Related Disturbances of Cholinergic Signaling in the Pathogenesis of AD and PD

The cholinergic system plays an important role in AD pathophysiology. The degeneration of cholinergic neurons of the basal forebrain in the early stage of AD and the accompanying decline in memory and cognitive functions have become the basis for the formulation of one of the oldest theories of the etiology of AD – the cholinergic hypothesis (Davies and Maloney, 1976; Whitehouse et al., 1981, 1982; Bartus et al., 1982; Wilcock et al., 1982; Ferreira-Vieira et al., 2016). The sites of the greatest concentration of neurofibrillary tangles made of aggregated tau protein in AD are the axons of cholinergic neurons projecting from the basal forebrain into the cortex. The presence of neurofibrillary tangles is accompanied by abnormal changes in the morphology and function of cholinergic neurons (Hampel et al., 2019). Changes in the cholinergic system during progression of AD have been documented by assessing the major functional components of cholinergic cells and signaling: the acetylcholine-synthesizing and -degrading enzymes, ChAT and acetylcholinesterase (AChE), respectively, VAChT that transports ACh into the vesicles, mAChRs and nAChRs, and the requirement of cholinergic neurons to receive neurotrophic support by NGF mediated by high- (TrkA) and low-affinity (p75^NTR^) receptors for survival (Hampel et al., 2018).

#### Degeneration of BFChN in AD

Studies on patients with AD have shown that in the early stages of the disease, the lesion involves mainly the presynaptic parts of the cholinergic system. Degeneration of cholinergic neurons manifests first as a dystrophy of NBM and MS axons, which are projecting to the cerebral cortex and hippocampus, respectively (Lehéricy et al., 1993; Hampel et al., 2018). In general, the number of BFChN in AD patients was reduced to about 40% of the control value, whereas the population of large cholinergic neurons was reduced by as much as 80%. In addition, morphometric measurements showed significant shrinkage (perikaryon diameter reduction by 80%) of surviving neurons (Rinne et al., 1987; Vogels et al., 1990). In the basal forebrain of murine models of AD, a significant loss of cholinergic neurons compared with control groups was also observed. The significant BFChN degeneration in mice matches that observed in post-mortem brains of patients with AD (Belarbi et al., 2009; Cranston et al., 2020). Studies have shown that abnormalities in cortical cholinergic axons, such as thickening or ballooning of terminals, are present in young people, but they are more frequent in non-demented elderly people. In severe AD, there is a decrease in the number of cholinergic axonal abnormalities relative to the mild form of AD, which could be the result of degeneration of axons with morphological abnormalities (Geula et al., 2008).

#### Aberrant Cholinergic Signaling Exacerbates Pathological Changes in AD

Reduction in ChAT activity (Davies, 1976; Perry et al., 1977; Rossor et al., 1982; Wilcock et al., 1982), cholinergic receptor number (Nordberg and Winblad, 1986), AChE activity (Davies, 1976; Geula and Mesulam, 1995), and decrease in high affinity choline uptake have been observed in AD (Rylett et al., 1983; Pascual et al., 1991). These changes are mainly the result of significant depletion of cholinergic axons in the cerebral cortex and the loss of cholinergic neurons in the basal forebrain (Geula and Mesulam, 1995).

Research on patients with AD has shown a decline in nAChR binding relative to older non-demented people in those cortical areas that are affected in AD, such as the medial temporal, insular, and posterior cingulate cortices (Sultzer et al., 2017). The impairment of the cholinergic α4β2 nAChR correlated with the greater amount of amyloid deposition (Okada et al., 2013) and impairment of cognitive functioning, especially episodic memory and executive function/working memory (Sabri et al., 2018) in patients with AD.

Studies on cell cultures and in guinea pig brains after systemic administration of physostigmine have shown that Ach may suppress Aβ production through stimulation of the non-amyloidogenic cleavage of APP by activation of mAChRs (Wolf et al., 1995; Beach et al., 2001) and nAChRs in PC12 cell culture (Kim et al., 1997). Furthermore, ACh has an impact on the phosphorylation of tau by mAChRs and nAChRs. Administration of nicotine or nAChR agonists to SH-SY5Y cell cultures overexpressing α3 or α7 nAChRs increased the level of phosphorylated and non-phosphorylated tau. This effect was reversed by nAChR antagonists (Del Barrio et al., 2011). In ApoE-deficient mice (murine model of familial AD), the administration of the M1 agonist caused a decrease in hyperphosphorylated tau level (reviewed in Fisher et al., 1998). Moreover, stimulation of muscarinic receptors led to the protection of cells from apoptosis caused by DNA damage, mitochondrial impairment, and oxidative stress in SH-SY5Y cells (Christopoulos and Mitchelson, 2003; De Sarno et al., 2003). There was evidence that Aβ causes the uncoupling of M1 mAChR from G-protein, which led to inhibition of the M1 receptor function in AD. These abnormalities may induce excessive production of Aβ by inhibition of signaling in the sAPPα pathway (Jiang et al., 2014).

#### Insufficient Neurotrophic Support in AD

Alterations in the NGF ability to interact with its two receptors, TrkA and p75^NTR^, have been observed in an AD mouse model, accompanied by a reduced concentration of matured NGF (mNGF) in the cortex and hippocampus (Capsoni et al., 2010). Significant differences in TrkAmRNA concentration in BFChN have been noted in patients with AD compared with age-matched controls (Mufson et al., 1996). No notable changes have been observed in the expression of p75^NTR^, suggesting that a selective deficit in TrkA signaling may be responsible for the reduced trophic support to BFChN. NGF is synthesized as the precursor protein pro-NGF. In AD, pro-NGF has been found to accumulate in the hippocampus and cortex (Fahnestock et al., 2004). A similar observation in Fisher 344 rat slices and in adult male Wistar rats with pharmacologically induced failure in NGF maturation implies a general shift in NGF metabolism, which may be responsible for the change in pro-NGF to NGF ratio (Bruno and Cuello, 2006; Allard et al., 2012). It has also been observed that the decrease in TrkA receptors is accompanied by reduced mNGF availability, suggesting that reduced trophic support may be responsible for the lower expression of the BFChN cholinergic phenotype (Allard et al., 2012). Pro-NGF signaling in the absence of mNGF has been shown to be capable of inducing atrophy of the BFChN and impairing memory consolidation in mice (Capsoni et al., 2011). In support of this, pharmacologically induced chronic failure in NGF maturation has been shown to result in increased pro-NGF level, cholinergic degeneration, and cognitive impairment in rat models (Allard et al., 2018). Cognitive deficits that arise from an increased pro-NGF signaling are a consequence of the interaction between pro-NGF and p75^NTR^ in the absence of its rival signaling partner mNGF. When p75^NTR^ is stimulated without concomitant TrkA signaling, it mediates a variety of intracellular cascades, leading to apoptosis.

#### Impairment of the Cholinergic System Is Involved in the Etiology of PD

Studies have shown that the cholinergic function of the basal forebrain is impaired in patients with PD (Arendt et al., 1983; Whitehouse et al., 1983). Loss of cholinergic cells in NBM is greater than that seen in AD (Liu et al., 2015). Positron emission tomography (PET) studies on PD and on Parkinson’s dementia showed cortical (Hiraoka et al., 2012; Shimada et al., 2015) and thalamic (Kotagal et al., 2012) reductions in AChE activity. In addition, AChE activity is lower in patients with PD with dementia than in patients with non-demented PD (Ruberg et al., 1986). Moreover, in patients with PD with or without dementia, reduced VAChT level in the parietal and occipital cortices (Kuhl et al., 1996), decreased nAChR binding and activity (Fujita et al., 2006; Oishi et al., 2007; Meyer et al., 2014), and increased mAChR binding in the frontal cortex (Asahina et al., 1998) were reported. Compared with controls, ChAT activity is decreased in patients with PD in the frontal cortex and the substantia innominata, and these changes correlate with the severity of dementia (Dubois et al., 1983). The cognitive decline observed in PD is also connected with cholinergic disturbance. It is even suggested that cortical cholinergic function is more severely affected in parkinsonian dementia than in AD (Bohnen et al., 2003). Moreover, cholinesterase inhibitors, such as rivastigmine and donepezil, exert beneficial effects on cognition and other behavioral symptoms (Mamikonyan et al., 2015).

There is evidence that degeneration of cholinergic neurons in the pedunculopontine nucleus may be connected with freezing of gait and postural instability. The number of AChE-positive neurons was reduced in patients with postural disability compared with those without balance deficits (Karachi et al., 2010). Patients with episodes of fall had lower VAChT expression in the thalamus than patients without episodes of fall. Similarly, patients with episodes of freezing showed a reduction of VAChT binding in the striatum and limbic archicortex (Liberini, 1997; Bohnen et al., 2019).

In summary, age-related deterioration in the functions of the cholinergic system is not particularly severe. It mainly concerns the attenuation of the phenotype of cholinergic neurons, which is manifested by a decrease in ACh secretion, moderate reduction of ChAT and VAChT levels, and lower expression of muscarinic receptors. In contrast, the development of neurodegenerative diseases is associated with very serious damage to the structure and functions of the cholinergic system, which is manifested by degeneration of cholinergic neurons, substantial loss of ChAT and AChE activity, a significant reduction in VAChT level, decreased ACh release, mAChRs and nAChRs signaling damage, reduced choline re-uptake, and lower neurotrophic support by NGF (Figure 2B).

## Dopaminergic System

### Involvement of Dopamine and Dopaminergic Receptors in the Regulation of Motor Function

In early 1960, because of the newly implemented method of formaldehyde histofluorescence, Carlsson, Falck, and Hillarp were the first to identify two novel catecholamines in the brain: noradrenaline and DA (Carlsson et al., 1962; Falck et al., 1962). In 1964, 12 different catecholaminergic cell groups were identified in the brain, located from the medulla oblongata to the hypothalamus (Dahlstroem and Fuxe, 1964). Later, another five cell groups were discovered in the diencephalon, olfactory bulb, and retina (Vogt Weisenhorn et al., 2016). DA is a catecholamine synthesized in the cytoplasm of dopaminergic neurons in the CNS from L-tyrosine. L-tyrosine in the presence of cofactors is transformed to L-DOPA by tyrosine hydroxylase (TH). Then, L-DOPA is then rapidly decarboxylated to dopamine by the aromatic L-amino acid decarboxylase (Musacchio, 2013).

Dopaminergic neurons are anatomically and functionally heterogeneous groups of cells. Nine dopaminergic cell groups were distinguished in the mammalian brain using methods of immunohistochemistry (Björklund and Dunnett, 2007). They are localized in the diencephalon, mesencephalon, and olfactory bulb. The nigrostriatal, mesolimbic, mesocortical, and tuberoinfundibular pathways are the main dopaminergic pathways in the brain (Figure 1). These pathways consist of cell bodies and axonal projections arising primarily from the SN, VTA, and the arcuate nucleus of the hypothalamus (Chinta and Andersen, 2005).

#### Motor System

The motor system integrates multimodal sensory information for posture maintenance and coordinated voluntary movement (Guyton and Hall, 2000; Takakusaki, 2013). Skeletal muscles are stimulated by motoneurons of the spinal cord. The activity of these neurons is controlled by motor centers in the cerebral cortex, pons, and brainstem (Kandel et al., 1991). Moreover, motor centers of the cerebral cortex and the spinal cord are controlled by the cerebellum and subcortical nuclei (Kandel et al., 1991; Purves et al., 2018).

Movement control is carried out through a complex, hierarchically ordered neural network. The cerebral cortex is involved in the control of voluntary movements and the motor decision process. Thalamus and basal ganglia (BG) use different pathways to initiate (direct pathway) and terminate (indirect pathway) the motor program by controlling muscle tone, muscle length, speed, and strength of the movement. Brainstem is responsible for postural control and regulation of muscle strength and tension. Interactions between the spinal cord and the structures of the cerebral cortex occur through the midbrain locomotor, subthalamic locomotor, and cerebellar locomotor regions (Takakusaki, 2013, 2017).

Basic locomotor pattern is generated by spinal interneural networks, referred to as the central pattern generator (CPG). This network generates rhythmic signals that cause stimulation of the antagonistic flexor and extensor muscles and promotes coordinated work of the limbs both in animals and humans. The activity of the CPG network is modified by direct or indirect modulation in the corticospinal tract and descending tracts of the brainstem (Takakusaki, 2013, 2017).

#### Receptors

Dopamine receptors are commonly expressed in the CNS, but they are also found in blood vessels, retina, heart, kidneys, and adrenals controlling the release of catecholamines and renin–angiotensin system (Missale et al., 1998; Ayano, 2016).

There are five main types of DA receptors. The dopamine 1 (D1) and dopamine 2 (D2) receptors are the most abundant receptors of all dopaminergic receptors in the CNS. D1 receptors are involved in the regulation of the release of neurotransmitters, such as GABA, glutamate, and ACh. D2 autoreceptors are found in both somatodendritic and axonal compartments and have a key role in the regulation of secretion in dopaminergic neurons. They control the timing and amount of DA released from their terminals in target regions in response to changes in the extracellular level of the neurotransmitter, as found in mice and rats (George and O’Dowd, 2007; Yapo et al., 2017; Marcott et al., 2018). It is believed that the activation of D2 presynaptic receptors is the basic mechanism of regulating DA release *via* a G-protein coupled receptor-mediated negative feedback loop (reviewed by Elsworth and Roth, 1997). Stimulation of D2 autoreceptors in mice and rats leads to a decrease in the concentration of cAMP and to a reduction in the phosphorylation of TH by protein kinase A (PKA) that, in turn, leads to reduced DA synthesis and decreased locomotor activity (Kebabian et al., 1972; Bao et al., 2010). *In vitro* research has shown that D2-type receptors may inhibit voltage-gated Ca^2+^ channels and that this could have a direct impact on DA release (Congar et al., 2002; Kisilevsky and Zamponi, 2008).

Almost all dopaminergic neurons express D3 receptors. They function as autoreceptors and regulate DA secretion in both ventral and dorsal striata. In mice, D3 receptors also inhibit DA release from presynaptic terminals; however, their contribution is significantly smaller compared with D2 receptors (Joseph et al., 2002). D4 receptors are highly distributed in frontal lobe regions; therefore, they are involved in the modulation of cognitive functions, whereas D5 receptors are expressed in postsynaptic dopamine-stimulated cells of the hypothalamus (Neve et al., 2004).

#### Basal Ganglia and Medium Spiny Neurons in the Striatum

Basal ganglia are a group of neuronal circuits, which are situated deep within the cerebral hemispheres. BG are strongly interconnected with the cerebral cortex, thalamus, and brainstem, as well as several other brain areas (Blandini et al., 2000). BG have many incompletely understood functions related to cognition and emotions, but they are best known for their role in movement control. The main components of BG are the corpus striatum (both ventral and dorsal parts), the globus pallidus [both internal (GPi) and external (GPe) segments], SN, and the subthalamic nucleus (STN) (Carpenter, 1976; Mas et al., 2017; Fazl and Fleisher, 2018; Young et al., 2020). The striatum is the main part of the neuronal circuits of BG. Caudate and putamen are partly separated by corticofugal and corticopetal fibers of the internal capsule (Lanciego et al., 2012).

Around 95% of neurons in the striatum are medium spiny neurons (MSNs) (Kemp and Powell, 1971), and they use GABA as a neurotransmitter. The remaining 5% of neurons in the corpus striatum are interneurons containing ACh, somatostatin, nicotinamide adenine dinucleotide phosphate (NADPH)-diaphorase, or GABA associated with parvalbumin, calretinin, or nitric oxide synthase (NOS) (Kawaguchi et al., 1995). In the striatum, besides dopaminergic inputs from SN, dopaminergic inputs from the cortical areas are also found.

Striatum exhibits a variety of receptors because of multiple inputs of diverse neurotransmitter systems (Sulzer et al., 2016; Jamwal and Kumar, 2019). There are two major pathways emerging within the BG, a direct excitatory pathway and an indirect inhibitory pathway. In the direct pathway, D1 receptor-expressing neurons in the striatum provide GABAergic axons to the GPi and to the SN pars reticulata. The GPi sends numerous inhibitory endings to the thalamus-modulating activity of the thalamic nuclei, which in turn regulates the activity of the motor cortex. In the indirect pathway, D2 receptor-containing striatal neurons send inhibitory GABAergic axons to the GPe, which in turn sends an inhibitory projection to the STN. The STN exerts a stimulating effect on the GPi inhibiting the thalamus and motor cortex (Lanciego et al., 2012; Freeze et al., 2013). The direct pathway is thought to facilitate movement while the indirect pathway suppresses movement. Dopaminergic signaling is crucial to the maintenance of physiological processes, and an unbalanced activity of dopaminergic pathways may lead to dysfunction related to neurodegenerative diseases (Milardi et al., 2019).

### Physiological Aging of the Dopaminergic System

Both human and animal studies indicate that the dopaminergic system components deteriorate during aging (Joyce, 2001; Branch et al., 2016). The increase in DA synthesis and the decrease in the number of dopaminergic receptors during aging depend on individual differences in vulnerability to nervous system damage (Reeves et al., 2002; Berry et al., 2016).

#### Changes in the Number of the Dopaminergic Neurons

The loss of dopaminergic neurons with age was observed in SN of mice (Noda et al., 2020) and humans (Ma et al., 1999; reviewed by Reeve et al., 2014); while in other structures, the number of dopaminergic neurons was not significantly reduced (Mann et al., 1984; Fearnley and Lees, 1991). An age-related loss of cells in the dopaminergic system in rats has been demonstrated (Sabel and Stein, 1981; Gao et al., 2011). The study of Felten et al. (1992a, b) in 26-month-old Fischer 344 rats revealed a 27% loss of DA cell bodies in SNpc and a 30% loss of DA nerve terminals in the rostral caudato-putamen, compared with 3-month-old control rats. They, however, mentioned that the absence of phenotypic markers for dopaminergic neurons does not necessarily mean that the cells have died. Aging may influence neurotrophic factor signaling and the regulation of tyrosine hydroxylase activity (Parkinson et al., 2015). Factors that contribute to neuronal loss in SN are oxidative stress, Ca^2+^ handling, respiratory deficiency, iron accumulation, or mitochondrial DNA defects. All of these factors may contribute to the development of PD (Hindle, 2010; Reeve et al., 2014).

In humans, the brain dopaminergic system also deteriorates with age. In SN, loss of dopaminergic neurons may reach up to 10% per decade (Ma et al., 1999; Buchman et al., 2012), which may correlate with motor and cognitive impairment (Mukherjee et al., 2002; Nyberg and Bäckman, 2004).

#### DA Metabolism, Receptors, and Oxidation

The results of a study on C57BL/6NNia mice revealed that morphological changes in dopaminergic neurons observed in 10-month-old adult animals were characterized by the accumulation of lipofuscin in dopaminergic neurons, a markedly reduced dopamine content, and an increase in the number of fragmented axons in the nigro-striatal pathway. These changes intensified until at least 30 months of age (McNeill et al., 1984).

Studies on rats indicate that there is age-dependent reduction in the level of DA release (Shimizu and Prasad, 1991; Gordon et al., 1995), DA uptake (Hebert and Gerhardt, 1999), DA transporters levels, and level of D2 receptors in postsynaptic sites in the striatum (Han et al., 1989; Roth and Joseph, 1994). Moreover, the mRNA level of DA transporter in the substantia nigra is reduced (Himi et al., 1995). These data suggest deterioration of DA release and uptake in the brain with age. Several studies have reported an age-related decline in DA concentration in the striatum (Cruz-Muros et al., 2007), but many other studies have not found such a decrease (Morgan and Finch, 1988; Felten et al., 1992b; Hebert and Gerhardt, 1999). A similar discrepancy has been noted in reports on DA receptor binding and DA uptake. However, most reports suggest an age-related decline in the DA nigrostriatal system in 24- to 27-month-old Fischer 344 and Sprague-Dawley rats (Strong et al., 1982, 1984; Marshall and Joyce, 1988; Felten et al., 1992b).

Human studies by PET and SPECT revealed that D1 and D2 receptors (Volkow et al., 1996; Kaasinen et al., 2000; Ishibashi et al., 2009; Karrer et al., 2017) and DA transporters (Volkow et al., 1996; Ishibashi et al., 2009; Karrer et al., 2017) are decreased in the nigrostriatal pathway. Interestingly, DA synthesis seems to be not affected or even enhanced (Kish et al., 1995; Braskie et al., 2008; Dreher et al., 2008); and since DA reuptake is lowered, DA may remain longer in the synaptic cleft in older individuals (Berry et al., 2016; Karrer et al., 2017).

Neuromelanin is a dark pigment appearing in several neuronal populations, mainly in dopaminergic neurons of SN. The formation of neuromelanin has been debated: whether it is enzymatically mediated or whether it is an autooxidation process of dopamine derivatives (Zecca et al., 2001). The level of neuromelanin increases with age before declining after 80 years of age (Zecca et al., 2002; Zucca et al., 2017; Carballo-Carbajal et al., 2019; Vila, 2019). It has been proposed that the synthesis of neuromelanin and its accumulation with age indicate an ongoing damage of cytosolic DA. Such a claim is supported by the fact that in dopaminergic neurons of VTA, only a small amount of melanin is generated over a lifetime. This may be related to the greater level of cytosolic dopamine in SN neurons as compared with VTA ones, which in turn may be related to the compensating activity of dihydropyridine-sensitive Ca_v_1.3 channels in SN but not VTA neurons (Mosharov et al., 2009). Increase in the level of dopamine-derived species disturbs the redox balance and escalates the oxidative stress during a period in life in which dementia syndromes develop (Sulzer et al., 2008).

Another area of damage due to neuromelanin accumulation may be the ubiquitin-proteasome system and intracellular vesicular trafficking. The equilibrium between dopamine and neuromelanin is crucial for cellular homeostasis; however, when neuromelanin-containing organelles accumulate high load of toxins and iron during aging, a neurodegenerative process can be triggered (Liang et al., 2004; Zucca et al., 2017). When neuromelanin takes up over 50% of the cytoplasmic volume of DA neurons about the sixth decade of life, this pigment potentiates the tendency of α-synuclein to form toxic protofibrillar and fibrillar species leading to cell degeneration (Shtilerman et al., 2002; Uversky et al., 2002). α-Synuclein plays a role in proteasome function, whose impairment can lead to the death of catecholaminergic neurons. The findings suggest that α-synuclein aggregation is a key feature associated with a decline in the proteasomal and lysosomal function and support the hypothesis that cell degeneration in PD involves dysfunction of these activities, impaired protein clearance, protein accumulation, and aggregation leading to cell death (Petrucelli et al., 2002; Xu et al., 2002; Zucca et al., 2018).

### Age-Related Disturbances of Dopaminergic Signaling in the Pathogenesis of PD and AD

The neurodegenerative process leading to the development of PD symptoms may last for many years (Hou et al., 2019). Deterioration of the dopaminergic system is believed to be the main cause of PD motor symptoms. The nigrostriatal pathway seems to be the most affected, but the mesolimbic and mesocortical pathways also appear to be affected (Vallone et al., 2000; Prediger et al., 2014). Deterioration of motor functions is associated with prominent loss of dopaminergic neurons in SN (Fearnley and Lees, 1991; White et al., 2010), the decline in striatal DA transporters levels (Miller et al., 1997), and reduction in striatal TH-positive axonal staining (Ryoo et al., 1998).

It should be added that in Parkinson’s disease, serious symptoms unrelated to motor impairment, such as anxiety, depression, cognitive decline, fatigue, disturbances of smell, sleep, and mood, and gastrointestinal function, are also observed. Non-motor symptoms may precede PD pathology by five or even more years, and at the beginning, they may not be linked with PD (Goldman and Postuma, 2014; Zhang et al., 2016). These symptoms are associated not only with damage of the dopaminergic projection but also with impairment of other neurotransmission pathways, such as those of glutamatergic and cholinergic, for example.

#### Loss of Dopaminergic Neurons in PD

Neuronal loss in PD arises as a result of oxidative stress, dysfunction of mitochondria (Bose and Beal, 2016; Segura-Aguilar, 2019; Valdinocci et al., 2019; Kim et al., 2021), deterioration of protein degeneration, and accumulation of α-synuclein or neuromelanin in SN (Reeve et al., 2014). Post-mortem brain samples obtained from patients with PD show that dopaminergic neurons are damaged because of mitochondrial dysfunction and chronic ROS production (Dauer and Przedborski, 2003). Mitochondrial abnormalities linked to the disease include mitochondrial electron transport chain impairment, alterations in mitochondrial morphology and dynamics, mitochondrial DNA mutations, and anomaly in Ca^2+^ homeostasis (Subramaniam and Chesselet, 2013). Substantial increase in oxidative stress and disruption of Ca^2+^ homeostasis and sustained increases in cytosolic-free Ca^2+^ were observed in several forms of PD. The etiology of PD involves defects in the mitochondrial respiratory chain (Perier et al., 2010, 2012), which in turn results in increased apoptotic cell death (Lev et al., 2003). The activity of complex I, which is one of the components of the mitochondrial electron transport chain, has been reported to be reduced in parkinsonian dementia with Lewy body disease (Poirier et al., 1994). Infusion of 1-methyl-4-phenyl-1,2,3,6- tetrahydrodropyridine (MPTP), which produces the animal model of PD by specific degeneration of dopaminergic neurons, selectively inhibits mitochondrial complex I (Langston et al., 1983; Burns et al., 1985).

#### Disturbances of Trophic Support in PD

Loss or disturbance of specific trophic factors, their receptors, or their signal cascades may also promote PD (Palasz et al., 2019a, 2020). Decreased levels of neurotrophic factors, such as BDNF (brain-derived neurotrophic factor), NGF, neurotrophin-3, and neurotrophin-4 were observed in PD (Siegel and Chauhan, 2000). BDNF, mesencephalic astrocyte-derived neurotrophic factor (MANF), glial cell line-derived neurotrophic factor (GDNF), and cerebral dopamine neurotrophic factor (CDNF) have been shown to be neuroprotective and neurorestorative toward damaged dopaminergic neurons in cell cultures and in various PD animal models (Allen et al., 2013; Voutilainen et al., 2015; Lindahl et al., 2017). Restoring the physiological level of these trophic factors by inducing their expression may be considered a therapy, which may halt the decline or even restore the function of the dopaminergic system (Langston, 2006; Palasz et al., 2017).

#### Non-motor Disturbances Related to the Dopaminergic System in PD

DA is involved not only in the regulation of movement control, but also contributes to the reward system, behavior, and learning (Schultz, 1998). In the reward pathway, dopaminergic neurons in the VTA project to the nucleus accumbens (NAc) and prefrontal cortex forming the mesocorticolimbic circuit (Cooper et al., 2017; Pavăl, 2017). DA can stimulate dopaminergic receptors located on MSN, which are the dominant cell type in NAc. Dopaminergic stimulation of MSN in the NAc core appears to be crucial for selecting motivational stimuli connected with reward or aversion (Soares-Cunha et al., 2020), while in the NAc shell this stimulation drives behavioral responses to repeated exposure to rewarding experiences (Meredith et al., 2008; Cooper et al., 2017). Age-related dysfunctions in the midbrain dopaminergic regulation of the human reward system were observed by PET examination (Dreher et al., 2008). Marked loss of dopaminergic system function and pathologies of reward stimuli processing were demonstrated both in healthy aging and in age-related neurodegenerative disorders, such as PD and AD, both in humans (Chau et al., 2018; Drew et al., 2020) and animal model studies (Ouachikh et al., 2013; Nobili et al., 2017).

The mesocorticolimbic ascending dopaminergic pathways to the prefrontal cortex are implicated in cognitive control of working memory and reward-based learning (Cools et al., 2019; Ott and Nieder, 2019; Douma and de Kloet, 2020). However, in many dopamine-related disorders, cognitive deficits are also accompanied by abnormal dopamine signaling in the striatum, which has been associated with impaired value-based learning, choice, and motivation. Similar findings from studies with experimental animals show that reversal learning is altered by damage to the ventral striatum, specifically the NAc (Nonomura and Samejima, 2019; Smith et al., 2020) or modulation of dopaminergic incoming signaling to the NAc (Alsiö et al., 2019). It has also been shown that dopaminergic receptors of the D1 and D2 families assume complementary roles in the control of cognitive processes (Bezu et al., 2017; Horst et al., 2019). Cognitive deficits in PD include structural, functional, and metabolic correlates. A distinction should be made between executive dysfunction mediated by dopaminergic transmission, seen in milder stages of PD compared with global dementia syndrome, which may occur as the disease progresses. In advanced PD, the deterioration of the neurotransmitter systems goes beyond the dopaminergic system and also involves the noradrenergic, serotonergic, and cholinergic systems, which are mutually responsible for the decline in cognitive ability (reviewed by O’Callaghan and Lewis, 2017).

Several psychological symptoms are associated with depression in patients with PD (Garlovsky et al., 2016). Depression is one of the most common non-motor symptoms related to PD, occurring in approximately 20% of patients. In many patients with PD, depression is the presenting symptom (Ryan et al., 2019). Magnetic resonance imaging showed changes in connections and decreased projection between the cortex and amygdala in patients with PD with depression. The amygdala is innervated by dopaminergic neurons originating from the VTA (Remy et al., 2005). Dysfunction of mesolimbic dopaminergic pathways derived from VTA and projecting to the amygdala and bed nucleus was reported (Volkow et al., 2011; Bananej et al., 2012; Hu et al., 2015; Huang et al., 2015). A number of studies also indicate the involvement of dopaminergic D1 and D2 receptors on the MSN of the NAc in the development of depression in patients with PD (Francis and Lobo, 2017).

#### Impairment in the Dopaminergic System Also Occurs in AD

Even though the dopaminergic system is not a key player in AD, it is affected in AD (Joyce et al., 1997; Martorana and Koch, 2014; D’Amelio et al., 2018). Although the loss of dopaminergic neurons has been identified in the AD brain (Roostaei et al., 2017), no neuronal loss was observed before the formation of Aβ plaques (Nam et al., 2018). In addition, a recent study connects the polymorphism of dopamine beta-hydroxylase with AD pathology (Belbin et al., 2019). Moreover, treatment with levodopa improved cognitive ability in patients with AD (Martorana et al., 2009). A recent meta-analysis linking the dopaminergic system and AD has summarized that the level of DA and D1 and D2 receptors were decreased in patients with AD (Pan et al., 2019). However, the role of this neurotransmitter system still remains unclear in AD.

Summing up, the changes in the dopaminergic system associated with aging mainly involve the weakening of the neurotransmitter phenotype of neurons and, thus, the deterioration of proper movement control. In contrast, in PD and other dementias with parkinsonian symptoms, a number of dopaminergic disorders are observed, such as increased susceptibility of DA neurons to toxic insults, substantial loss of DA neurons in SN and VTA, significant reduction of dopaminergic projection in the striatum, a severe decline in striatal dopamine transporter level, polymorphism of dopamine β-hydroxylase, accumulation of neuromelanin and β-synuclein in DA neurons of SN, and loss of trophic support specific for dopaminergic neurons by GDNF, BDNF, and CDNF.

In general, a comparison of brain changes within the glutamatergic, cholinergic, and dopaminergic systems in healthy elderly people and in people who exhibit symptoms of neurodegenerative diseases shows that there are fundamental morphological, biochemical and functional differences between normal physiological aging and pathological aging associated with disease processes, the most common of which are the so-called dementia syndromes. These disease processes are usually irreversible and result in severe disturbances in the three projection systems discussed (Figure 2).

## Therapeutic Applications

The search for an AD therapy appears to be an extremely frustrating endeavor. Liu et al. (2019) counted 2,117 clinical trials testing various approaches for the treatment of AD. As a result, no new drug for AD has been approved since the approval of memantine (an NMDAR antagonist) in 2003 following the approval in 2001 of galantamine, the last of four acetylcholine esterase inhibitors (AChEIs) (the others being tacrine, donepezil, rivastigmine) recommended in the treatment of dementia (Cummings et al., 2014). Both memantine and AChEIs are regarded as symptomatic drugs, although their postulated mechanism of action may confer neuroprotection.

Similarly, only symptomatic treatment with L-DOPA and agonists of dopamine receptors are presently available treatments for PD. There is no pharmacotherapy of PD that would stop or delay the progression of this disease (Fox et al., 2018; Hayes et al., 2019).

### Addressing Glutamatergic System

Memantine has moderate binding affinity and rapid blocking–unblocking receptor kinetics (Parsons et al., 1993; Danysz et al., 2000). Thanks to this binding profile, memantine may correct the dysfunctional NMDAR activation present in AD. Mild, but chronic stimulation of glutamatergic receptors may ultimately lead to neuronal damage/death. This low-level pathological stimulation is blocked by memantine, however, when a physiological signal arrives in the form of a transiently elevated glutamate level, and memantine dissociates from NMDAR allowing for normal neurotransmission. Accordingly, memantine allows the better detection of physiological stimuli and at the same time confers neuroprotection (Danysz et al., 2000; Parsons et al., 2007). Memantine may also protect neurons from the excitotoxicity caused by excessive activation of NMDARs (Willard et al., 2000).

It is likely that modification of memantine leading to its enhanced binding to extra-synaptic NMDA receptors will be beneficial. These receptors are localized on dendrites and non-perisynaptic parts of the dendritic spines, and their activation requires a high glutamate level (Hardingham et al., 2002). Bordji et al. (2010) found that sustained activation of extra-synaptic NMDARs but not synaptic NMDARs strongly enhanced the neuronal production of Aβ. A kind of vicious cycle takes place when Aβ increases glutamate level, which in turn increases Aβ production. Thus, specific inhibition of extra-synaptic NMDARs seems a desired goal. Nitromemantine, an improved NDMAR antagonist, is able to selectively block the aberrant activation of extra-synaptic NMDARs and protects synapses from Aβ-induced damage both *in vitro* and *in vivo* (Talantova et al., 2013). However, it should also be noted that memantine reduces the levels of the secreted form of Aβ precursor protein (APP) and secreted Aβ in human neuroblastoma cells (Ray et al., 2010).

It is interesting that a single molecule may be a multi-targeting compound. Memantine is likely to be such a molecule, and Song et al. (2008) showed in rat primary cortical cultured neurons that memantine decreased tau phosphorylation, elevated by Aβ. Wang et al. (2015) demonstrated that memantine can rescue protein phosphatase-2A (PP2A) activity, inhibited by I_1_^PP2A^ – an endogenous inhibitor of this phosphatase. This inhibitor is upregulated in the AD brain, and its overexpression in Wistar rats, induced with a viral vector, resulted in abnormal hyperphosphorylation of tau and neurodegeneration. Memantine rescued PP2A activity by decreasing its demethylation at Leu309 selectively, which resulted in the inhibition of tau phosphorylation and prevented neurodegeneration. Of note, memantine exerted these effects by non-NMDAR-mediated interaction; thus, it may regard not only NMDAR antagonist but also an anti-tau-phosphorylation agent. In addition, memantine may upregulate autophagic flux, and this effect was found to be independent of its interaction with NMDARs (Hirano et al., 2019). This was inferred from the observation that neither agonist nor antagonist of NMDARs (NMDA and D-AP5, respectively), in the presence or absence of memantine, affected the level of LC3-II, in contrast to memantine alone, which elevated the LC3-II level. LC3-II is the microtubule-associated protein 1A/1B-light chain 3 (LC3) conjugated to phosphatidylethanolamine and serves as an autophagy marker. However, this observation does not exclude the possibility that memantine may also induce stimulation of autophagy *via* NMDARs. This possibility could not be verified by the authors of the study, as NMDAR may have been non-functional in the SHSY5Y cells used in this experiment. However, in the T-98G model, a malignant glioma cell line, the knockdown of NMDAR prevented memantine-induced increase of LC3-II (Yoon et al., 2017), thus in these cells, the presence of NMDAR seems indispensable for pro-autophagic effects of memantine.

With respect to the glutamatergic system, the dysfunctional activation of NMDARs may be caused by the impaired uptake of glutamate by astrocytes through their EAATs, mainly by EAAT2. In AD, the loss of EAAT2 protein and function is observed (Jacob et al., 2007). Aβ impairs the function of EAAT2 in glia, attenuating glutamate clearance in the synaptic cleft (Scimemi et al., 2013; Di Domenico et al., 2017). In addition, Aβ enhances the release of glutamate from presynaptic neurons (Brito-Moreira et al., 2011) and astrocytes (Talantova et al., 2013). Elevated glutamate level can cause a low level of excitation, which removes Mg^2+^ blockage of the NMDAR and increases the continuous influx of Ca^2+^ into postsynaptic neurons. This causes “background noise” at rest (Parsons et al., 2013), which interferes with the physiological activation of NMDARs. Recovery of EAAT2 function, impaired by Aβ, may help reduce the excessive resting level of glutamate. Two compounds were synthetized, which act as EAAT2 activators. They were able to enhance the glutamate translocation rate in cultured astrocytes (Kortagere et al., 2018). Both of these compounds may have therapeutic potential, but this has to be confirmed by in *vivo* studies.

### Addressing Cholinergic System

It is believed that the degeneration of cholinergic neurons observed in AD causes a decline in ACh level, which weakens signaling and leads to cognitive decline and memory impairment (Terry and Buccafusco, 2003). In advanced AD, the Ach level is decreased by 10% (Giacobini, 2003). Inhibition of the action of the ACh hydrolyzing enzyme AChE by AChEIs should elevate the level of ACh and in this way help to alleviate the symptoms of AD. Elevated ACh levels may also have neuroprotective effects, since they will increase NGF secretion. On the other hand, the lower level of ACh attenuates the production and release of NGF from the hippocampus and cortex, the projection targets for cholinergic neurons. This leads to diminished NGF uptake by presynaptic terminals of the cholinergic projection axons and finally to the deficit of retrogradely transported NGF in the BFChN. As a result, this deficit results in BFChN atrophy or in the loss of their cholinergic phenotype. This further leads to the decline in ACh level and a kind of vicious cycle ensues. Once the level of ACh is raised by an AChEI, the above-described process is reversed, and the chances for the continued survival of BFChN are increased (Cuello et al., 2019).

Treatment with AChEIs may be an appropriate line of therapy, but the question arises as to whether the treatment may be modified in order to increase its efficacy and target specificity. One interesting approach is presented by Moss (2020). His underlying thesis is the contention that the inhibition of AChE exerted by currently used AChEIs is too weak. This inhibition is dose-dependent, but it is not possible to increase daily doses of AChEIs because of gastric side effects caused by these drugs. He proposes to replace these short-acting compounds with irreversible AChEIs, which are long-acting and are inherently CNS selective. Such compounds are more than twice as effective in inhibiting brain AChE. Methane sulfonyl fluoride (MSF) is a well-known irreversible AChE inhibitor. When given orally over 8 weeks to patients with mild or moderate AD (Moss et al., 1999), it produced an estimated ∼66% inhibition of AChE in the brain, which improved cognition, an improvement that persisted unabated through the following 8 weeks after ending MSF. MSF itself is a compound with a short half-life in an aqueous environment, and a much higher degree of AChE inhibition in the CNS as compared with peripheral tissues derives entirely from the fact that the rate of AChE synthesis is much lower in the former than in the latter. As a result, during the necessary repeated MSF dosing, the AChE inhibition level stabilizes at a low level in the peripheral tissues while continues to grow in CNS. On one hand, it confers to MSF selectivity toward CNS; and on the other, it raises the danger of surpassing the upper limit of the therapeutic window.

Another approach is to activate α7 AChR. It was found (Medeiros et al., 2014) that this may have a pro-cognitive effect. A-582941, a selective α7 nAChR agonist, given to aged 3xTg-AD mice, restored their cognition to the level observed in age-matched non-transgenic mice. At the molecular level, A-582941 induced the expression of c-Fos and BDNF, and phosphorylation of CREB and neurotrophic tyrosine receptor kinase type 2 (NTRK2). However, the A-582941 treatment did not ameliorate AD-associated changes, such as Aβ deposits, tau phosphorylation, and the presence of inflammatory cells. In contrast, Wang X. L. et al. (2020) activated α7 nAChR in primary hippocampal cells and in a APPswe/PSEN1dE9 double-transgenic AD mouse model with PNU-282987. The activation of α7 nAChR improved the cognitive abilities of mice and, among others, reduced the deposition of Aβ in the hippocampus. Interestingly, Cecon et al. (2019) found that Aβ binds specifically and with high affinity to α7 nAChR and that this can be prevented and reversed with, among other compounds, the above-mentioned PNU-282987.

Rivastigmine, a drug mainly used in AD, is possibly useful as an adjunct therapy for general or specific motor symptoms including gait. It was found that the degree of cognitive impairment is strongly associated with the incidence of fall, which is a major problem for many patients with PD. Rivastigmine, by its action as an inhibitor of AChE, can slow the deterioration of cognitive function and lower the incidence of fall in these patients (Li et al., 2015). These findings are in line with the evidence of serious cholinergic denervation in PD (Bohnen and Albin, 2011).

### Combined Memantine and AChEIs Treatment

It seemed plausible that the combined treatment with memantine plus one of the AChEIs may increase the efficacy of the medication. Combined donepezil and memantine were approved as a treatment for patients with moderate to severe AD in 2014 (Deardorff and Grossberg, 2016). The combination was more beneficial for cognition, functioning, behavior, and global assessment than donepezil alone in patients with mild or severe AD (Tariot et al., 2004). A subsequent analysis of this study encompassing 404 patients revealed the superiority of combined therapy, among others, in language and memory (Schmitt et al., 2006), and the improvement or stabilization of symptoms occurred in a greater proportion of patients in the combination treatment group than in the donepezil only group (van Dyck et al., 2006).

It has been shown that most patients with moderate/severe AD with sustained cognitive decline on AChEI monotherapy stabilized or improved on the mini-mental state examination (MMSE) score with the addition of memantine (Dantoine et al., 2006). However, the advantages of combination therapy appeared to be limited to moderate/severe AD, as the inclusion of patients with mild AD and the omission of severe AD cases made the difference between patients treated with AChEIs (donepezil, rivastigmine, galantamine) in combination with memantine and the corresponding monotherapy groups insignificant (Porsteinsson et al., 2008). When, however, patients with mild AD were excluded from the subsequent analysis, the combination treatment regained its statistically significant superiority over AChEIs monotherapies (Atri et al., 2013). In this context, it should be noted that memantine is not approved for the treatment of mild AD.

Favorable outcome of combined therapies in moderate/severe AD was further confirmed when an extended-release formulation of memantine was added to treatment with AChEIs (Grossberg et al., 2013). The *post hoc* analysis of this clinical trial (NCT00322153) revealed that this formulation of memantine, when added to AChEI treatment, resulted in early and maintained improvement evaluated by neuropsychiatric inventory, clinician’s interview-based impression of change, and activities of daily living scales (Grossberg et al., 2018). In a trial (ISRCTN49545035), where donepezil was (1) continued, (2) discontinued, (3) replaced by memantine, and (4) continued with memantine added, the best results were obtained for the latter case, although the improvement was insignificant, maybe because of the small sample size. Interestingly, the authors concluded their study with the statement that the combination of donepezil and memantine brought no significant benefits over donepezil given alone (Howard et al., 2012).

A meta-analysis of 54 studies on placebo-controlled AD trials of donepezil and memantine alone or in combination, published up to early 2020, allowed to state that this combination showed better outcomes in cognition, global assessment, daily activities, and neuropsychiatric symptoms, when compared with monotherapy and placebo. Importantly, the inclusion of memantine slows the progression of AD (Guo et al., 2020). The following picture emerged based on meta-analysis of the results of 41 randomized controlled AD trials, published up to mid-2017, in which the effects of treatment of AD from mild to the severe stage with AChEIs and memantine were reported (Dou et al., 2018). It can be concluded that with respect to cognition, both galantamine and donepezil were probably most effective in mild/moderate AD, while donepezil plus memantine in moderate/severe AD. Neither of the treatments was likely to improve neuropsychiatric symptoms based on the 10-item version of the neuropsychiatric inventory (NPI) (delusions, hallucinations, agitation, depression, anxiety, euphoria, apathy, disinhibition, irritability, aberrant motor behavior), and the findings of Dou et al. (2018) diverged from those reported by Guo et al. (2020), who claimed that each treatment did improve these symptoms.

The effect of memantine in slowing down AD progression had been reported in a meta-analysis by Wilkinson and Andersen (2007), based on six placebo-controlled, 6-month studies in patients with moderate/severe AD. Two of these studies included AChEIs. Significantly more (28%) placebo-treated patients showed any clinical worsening than memantine-treated ones (18%), and that marked clinical worsening also occurred more frequently in placebo-treated patients compared with memantine-treated ones (21% vs. 11%). A more recent meta-analysis, based on 10 trials performed between 1997 and 2011 (Kennedy et al., 2018) brought rather discouraging results; and the authors found that patients receiving AChEI or memantine experienced a significantly greater annual rate of decline on Alzheimer Disease Assessment Scale-cognitive subscale (ADAS-cog) than patients receiving neither medication. It should be remembered that the higher ADAS-cog score, the worse the performance. However, it is a bit puzzling that in the table entitled, Participant Characteristics by Concomitant Medication Group the ADAS-cog score was the highest in the no medication group, and after 24 months it was still the highest in this group, concomitantly with the score rise, which was also the greatest in this group, more than twice of score rise in the other groups.

Yet another meta-analysis (McShane et al., 2019) reported that in moderate/severe AD (with or without AChEIs medication), memantine consistently offered a small clinical benefit versus placebo but brought no benefit in mild AD. Finally, Liang et al. (2018), by Bayesian network meta-analysis (NMA) for the analysis of MMSE results from 35 clinical trials, arrived with the conclusion that memantine ranked the highest from six AD drugs, i.e., memantine and five AChEIs with respect to cognitive benefit.

The effectiveness of memantine and AChEIs is slightly different when considering their effect on structural changes in the brain. Rate of brain volume changes, for instance, the annualized percent change of total brain volume (% TBV/y) may be seen as an objective measure of neuroprotection. In this regard, there is convincing evidence that treatment with AChEIs is more effective in slowing down this rate than memantine (Kishi et al., 2015).

In the face of some ambiguity, it is worth quoting McShane et al. (2019) conclusion of authors: Clinical heterogeneity in AD makes it unlikely that any single drug will have a large effect size, and means that the optimal drug treatment may involve multiple drugs, each having an effect size that may be less than the minimum clinically important difference.” A multi-target approach to AD treatment is being seen increasingly as viable. However, no matter how tempting this approach may be, its application requires adopting a fundamental criterion, namely, that the effect of combined drugs therapy should be greater than the effect of each of the drugs given alone. In light of the data discussed above, the fulfillment of this criterion has yet to be achieved.

### Addressing Dopaminergic System

In contrast to AD, where symptomatic treatments bring rather marginal effects, in PD, the characteristic motor impairments of PD can be relieved to some extent by treatment. Although impairments appear once the loss of dopaminergic neurons in SN is already advanced, symptomatic treatment for some time with levodopa preparations, DA agonists, and monoamine oxidase-B (MAO-B) inhibitors is clinically effective (Fox et al., 2018). The recommendations and descriptions of the vast repertoire of drugs available for the treatment of various aspects of PD are permanently updated, and the reader is referred to the many comprehensive studies that address this issue, e.g., Troncoso-Escudero et al. (2020).

Currently, no disease-modifying treatment of PD exists that would stop or delay the progression of this disease (Fox et al., 2018; Hayes et al., 2019). It is suggested that physical activity may be both preventive against PD and slow PD progression (Ahlskog, 2018), although confirmation by clinically relevant trials is lacking. This is in contrast to animal studies, where in many instances physical training efficiently ameliorated or completely abolished the effects of various insults against dopaminergic neurons (Palasz et al., 2019a). Studies revealed that physical exercise influenced multiple pro- and anti- neurodegenerative processes, specifically involving neurotrophic factors, notably BDNF (Palasz et al., 2019b, 2020).

### Deep Brain Stimulation Effects on Neurotransmitter Signaling

High-frequency electrical stimulation applied to chosen brain structures, named deep brain stimulation (DBS), turns out to be a highly effective therapeutic measure in many instances, notably in movement disorders. Initially, it was assumed that DBS silenced these structures, causing their functional lesion. For instance, DBS applied to STN may reduce rigidity, tremor, and bradykinesia, i.e., may reverse PD hypokinetic state. This is due to the reduction of glutamatergic output from STN directed to GPi, which attenuates GPi activity, resulting in reduced inhibition of the thalamus and alleviation of hypokinetic symptoms (Hashimoto et al., 2003). In a similar way, STN-DBS reduces the inhibition of thalamus exerted by SNpc, which is the other main target of STN.

One recent view is that electrical stimulation can entrain a less detrimental pattern of activity of dysfunctional neuronal circuits (McIntyre and Anderson, 2016). Such a view seems more compatible with the observations that DBS of GPi may alleviate hypokinetic symptoms in PD and hyperkinetic ones in dystonia (Jakobs et al., 2019).

Besides immediate symptomatic effects, DBS may also exert neurochemical effects (McIntyre and Hahn, 2010). For instance, Lee et al. (2006) found in rats that continuous electrical stimulation of the subthalamic nucleus evoked short-lasting increase of striatal DA efflux and that stimulation of the area dorsomedial to STN caused about 10 times greater DA efflux, which gradually reached a plateau and then slowly decreased. Various brain structures are electrically stimulated for specific purposes, for instance, DBS was applied to NBM with the intention of improving memory in patients with dementia (Mankin and Fried, 2020). Chronic stimulation of this nucleus resulted in slowing of cognitive decline as measured by ADAS-cog, ADAS memory, and MMSE, in a small group of early- and patients with late-stage AD (Kuhn et al., 2015). These results should be regarded with caution, a recent clinical trial provided Class II evidence that in patients with dementia with Lewy body disease, active DBS stimulation of NBM did not significantly change selective recall scores compared with sham stimulation (Maltête et al., 2021).

## Summary

The efficiency of cholinergic, glutamatergic, and dopaminergic projecting systems declines slowly during physiological aging because of a slow but progressive deterioration as a result of various mechanisms. The residual efficiency of these systems, however, allows the elderly to remain fully independent in daily activities and even to continue professional activities. It is likely that the accelerated worsening of various processes leads to severe and extensive impairments in their functioning, aggravated by the pathological processes, which are not observed in physiological aging. The overall efficiency of projecting systems declines gradually, diminishing functional independence and hampering professional activities. At present, there are no therapies that reverse or at least stop the progression of the demise of these systems. Thus, inevitably, functional regression continues, rendering those affected to end up on life-long, round-the-clock care.

## Conclusion and Outlook

Physiological aging of the nervous system does not significantly deteriorate the functions of neurotransmission systems in the brain, such as the glutamatergic, cholinergic, and dopaminergic systems. These projection systems remain functional during physiological aging and have the ability to compensate for adverse changes with age. However, the failure of these systems underlies specific aspects of dementia and motor disorders associated with Alzheimer’s and Parkinson’s diseases.

In the context of late-onset neurodegenerative diseases, we hypothesize that there are genetic or environmental factors that interfere with intrinsic measures supporting physiological aging. The key question is what has been changed by aging that renders the nervous system vulnerable to such genetic and environmental factors that make it finally succumb to fatal, irreversible neurodegeneration. Alternatively, we could ask how the nervous system maintains its normal aging. There are a number of inevitable, age-dependent alterations. Some of them may be inconsequential, the others detrimental, but because of compensatory strategies emerging during physiological aging, a sufficient level of functioning for a particular transmitter system may be maintained. This, in turn, leads us to ask why compensatory strategies lose their efficiency in neurodegenerative diseases.

The glutamatergic, cholinergic, and dopaminergic projection systems are the target of clinical pharmacotherapy with limited and transient efficacy in advanced stages of neurodegeneration. These neurotransmitter systems may also serve as good targets in prophylactic therapy, but this would need a detailed search for changes related to early aging and compensatory mechanisms seem justified. Introduction of early treatments may increase the chances of maintaining physiological aging of the nervous system despite the presence of genetic and/or environmental risk factors (Figure 2A). It is essential to find out if there is a time prior to neurodegeneration when prophylactic therapy maybe effective since, despite the many different initial causes of neurodegenerative disorders, the disease process leads to unstoppable and lethal neurodegeneration. It can be hypothesized that the transition from physiological aging to profound nervous system dysfunction in neurodegenerative diseases is caused by impaired compensatory processes and the emergence of self-aggravating or, more likely, mutually aggravating processes. If this proves to be true, it is imperative to identify these processes as they may be likely targets of pharmacological intervention, even in advanced stages of neurodegeneration.

## Author Contributions

GN, WN, and MS: conceptualization. MW, MZ, PD, MS, and AG: PubMed searching. WN, AG, and GN: validation. MW, PD, MZ, AG, WN, and GN: writing and original draft preparation. AG, MW, WN, and GN: the figure conceptualization and preparation. WN and GN: writing, review, and editing and funding acquisition. All authors: read and agreed to the published version of the manuscript.

## Conflict of Interest

The authors declare that this study was partially sponsored by TauRx Therapeutics Ltd. The funder was not involved in the paper design, collection, analysis, interpretation of reviewed literature resources, the writing of this article or the decision to submit it for publication.
